# Viral hepatitis in China during 2002–2021: epidemiology and influence factors through a country-level modeling study

**DOI:** 10.1186/s12889-024-19318-8

**Published:** 2024-07-08

**Authors:** Ning Sun, Fangli He, Jiufeng Sun, Guanghu Zhu

**Affiliations:** 1 School of Mathematics and Computing Science, Guangxi Colleges and Universities Key Laboratory of Data Analysis and Computation, Guilin University of Electronic Technology, Guilin, 541004 China; 2 Center for Applied Mathematics of Guangxi (GUET), Guilin, 541004 China; 3 Guangdong Provincial Institute of Public Health, Guangdong Provincial Center for Disease Control and Prevention, Guangzhou, China

**Keywords:** Viral hepatitis, JoinPoint model, Global principal component analysis, Panel fixed-effects modeling

## Abstract

**Background:**

Viral hepatitis imposes a heavy disease burden worldwide and is also one of the most serious public health problems in China. We aimed to describe the epidemiological characteristics of hepatitis in China and to investigate the influencing factors.

**Methods:**

We first used the JoinPoint model to analyze the percentage change (APC) and average annual percentage change (AAPC) of hepatitis in Chinese provinces from 2002 to 2021. We then explored the influencing factors by using the time-series global principal component analysis (GPCA) and the panel fixed-effects model.

**Results:**

The disease burden varied across different provinces from 2002 to 2021. The AAPC of the total HAV incidence decreased by 10.39% (95% CI: [-12.70%, -8.02%]) from 2002 to 2021. Yet the AAPC of HBV, HCV, and HEV increased by 1.50% (95% CI: [0.23%, 2.79%]), 13.99% (95% CI: [11.28%, 16.77%]), and 7.10% (95% CI: [0.90%, 13.69%]), respectively. The hotspots of HAV, HBV, HCV, and HEV moved from the west to the center, from the northwest to the southeast, from the northeast to the whole country, and from the northeast to the southeast, respectively. Different types of viral hepatitis infections were associated with hygiene, pollutant, and meteorological factors. Their roles in spatial-temporal incidence were expressed by panel regression functions.

**Conclusions:**

Viral hepatitis infection in China showed spatiotemporal heterogeneity. Interventions should be tailored to its epidemiological characteristics and determinants of viral hepatitis.

**Supplementary Information:**

The online version contains supplementary material available at 10.1186/s12889-024-19318-8.

## Introduction

Viral hepatitis, primarily caused by infection with five types of viruses (A-E), is a critically severe public health issue around the world [1]. Hepatitis A (HAV) and Hepatitis E (HEV) are transmitted by the fecal-oral route and are mainly acute infections. Hepatitis B (HBV) and Hepatitis C (HCV) are transmitted through sexual contact and drug injection, and are the most common causes of cirrhosis, cancer, and viral hepatitis-related deaths, with a greater likelihood of progression to chronic infection [2]. Hepatitis B and C cause 1.1 million deaths and 3 million new infections per year [1]. The World Health Organization’s global hepatitis strategy has been endorsed by all member states. Its goal is to reduce the number of new hepatitis infections by 90% and decrease the number of deaths by 65% between 2016 and 2030 [3]. China has made significant achievements in the prevention and control of viral hepatitis. However, due to its large population, China still carries a substantial burden of viral hepatitis [4]. Currently, China has about 87 million chronic carriers of HBV (constituting one-third of the global cases) and around 7.6 million chronic HCV infections, with large spatial-temporal variability in different provinces [5]. There is an urgent need to clarify the epidemiological characteristics of viral hepatitis in China.

Previous studies have shown that viral hepatitis infection is associated with the combined impacts of social, demographic, and meteorological factors, resulting in high spatiotemporal heterogeneity in prevalence [6, 7]. Meteorological factors (e.g. temperature and precipitation) can modify the transmission of viral hepatitis through various pathways [8]. Research conducted in China has found that temperature, precipitation, extreme rainfall, floods, and seasonal climate changes played different roles in the transmission of viral hepatitis, with a spatiotemporal clustering of viral hepatitis [9, 10]. Risk factors include male gender, old age, unhealthy lifestyles, low GDP, and living in rural or western regions [11, 12].

The present study went a further step to describe the overall disease burden of hepatitis in China, as well as the epidemiological characteristics and spatiotemporal distribution of hepatitis. The JoinPoint model was applied to analyze the APC and AAPC in hepatitis incidence rates across Chinese provinces from 2002 to 2021. A time-series global principal component analysis was conducted to analyze the influencing factors of viral hepatitis, followed by panel fixed-effects regression to assess the impact of these factors on different regions of China.

## Data and methodology

### Data sources

Morbidity data from 31 provinces, autonomous regions, and municipalities in China from 2002 to 2021, according to the categorization of hepatitis A-E, were obtained from the China Hygiene and Health Statistics Yearbook. Data on various indicators, including the number of beds in healthcare institutions (Beds), healthcare personnel (HCP), healthcare institutions (HCIs), annual precipitation, annual average temperature, annual average relative humidity, ammonia nitrogen emissions (NH3-N), chemical oxygen demand emissions (COD), and sulfur dioxide emissions (SO2), were extracted from the China Statistical Yearbook and the statistical yearbooks of various provinces covering the study period.

### JoinPoint model

The JoinPoint regression model was used to establish segmented regression based on the temporal characteristics of disease distribution [13]. It involved identifying several connecting points to divide the study period into different intervals. Trend fitting and optimization were then conducted for each interval to evaluate the disease change across different intervals. The main outcome indicators include APC, AAPC, and 95% confidence intervals (CI). Applying the JoinPoint model to fit the incidence rates of viral hepatitis in each province of China from 2002 to 2021, segmented trends of viral hepatitis across provinces in China were derived.

### Spatial autocorrelation and ANOVA multifactor analysis

We used global Moran’s I to assesses the overall spatial autocorrelation of a dataset to determine if data points tend to be similar across the entire study area. We further used local Moran’s I to identify local spatial clustering phenomena such as high-high or low-low clusters. Additionally, local indicators of spatial association (LISA) maps were derived from the results of local Moran’s I, providing a visualization and further analysis of significant clustering areas in the space. These tools are supplemented with p-values to statistically validate the identified spatial patterns.

Utilizing the ANOVA multifactor analysis in variance model, we investigated the differences in the incidence rate of viral hepatitis across China over 17 years. The Tukey multiple comparison method was employed to determine the differences. According to the economic zones in the statistical system and classification standards [14], for zoning analysis, the country was divided into the East (including Beijing, Tianjin, Hebei, Shanghai, Jiangsu, Zhejiang, Fujian, Shandong, Guangdong, and Hainan), the Center (including Shanxi, Anhui, Jiangxi, Henan, Hubei, and Hunan), the West (Inner Mongolia Autonomous Region, Guangxi, Chongqing, Sichuan, Guizhou, Yunnan, Tibet, Shaanxi, Gansu, Qinghai, Ningxia, and Xinjiang), and the four major regions of the Northeast (Liaoning, Jilin, and Heilongjiang).

### Time-series global principal component analysis

GPCA is an extended principal component analysis method designed for dealing with time-varying data series, which is more effective at capturing variability and patterns of the data. It considers the variance of both the overall dataset and each time point. Nine indicators and panel data were selected for GPCA in view of the influence factors of viral hepatitis (the main pollutant emissions in wastewater and exhaust gas, the level of health care in each province, and meteorological factors). The missing values of the number of beds in health institutions in 2008 were interpolated and standardized. The eigenvalues were calculated and the cumulative variance contribution ratio was derived by KMO and Bartlett’s test. Factors were determined based on the principle that the cumulative variance contribution rate was greater than or equal to 80%. After conducting factor analysis, three factors were named as: hygiene factor (i.e. the overall level of healthcare services and medical resources in each province), meteorological factor (i.e. meteorological conditions), and pollutant factor (i.e. the emissions of major pollutants in wastewater and exhaust gases). Attaining a high score in both the pollutant and hygiene factors signifies enhanced environmental and health conditions.

### Panel fixed effects model

Fixed effects modeling (FEM) is a panel data analysis method. It considers the dual dimensions of individual and time to study the relationship between variables under different individuals and time, while controlling individual fixed effects. Here based the panel data of 31 regions from 2005 to 2021, we first tested the set form of the panel model by using the F-statistic, and then applied the Hausman test to choose the use of fixed or random effect model, and we then employed the panel fixed effect regression. The main factors derived from GPCA were utilized to regress with the four types of hepatitis separately to produce panel regression results for the whole country and the four regions.

We also introduced a dynamic panel model and considered the lag structure of variables, aiming to enhance our capability to capture time-dependencies in the data. However, due to the short time span of our annual dataset, and because the introduction of lagged variables exacerbated endogeneity issues, some variables lost their statistical significance, thereby diminishing the explanatory power of the model. The potential correlation between lagged variables and the error term could lead to biased estimates, affecting the accuracy of the model. Therefore, given the technical and data limitations, we chose not to use a lag structure to ensure the robustness and reliability of our results, avoiding potential issues from insufficient data and model overfitting.

### Software tools

The JoinPoint regression model was conducted by JoinPoint Trend Analysis Software (version 5.0.2). GPCA analysis and panel fixed-effects modeling were operated by R software (version 4.2.1). R and PYTHON (version 3.8.5) software were used for drawing the plots.

## Results

### Trends of viral hepatitis in China

**Fig. 1:**
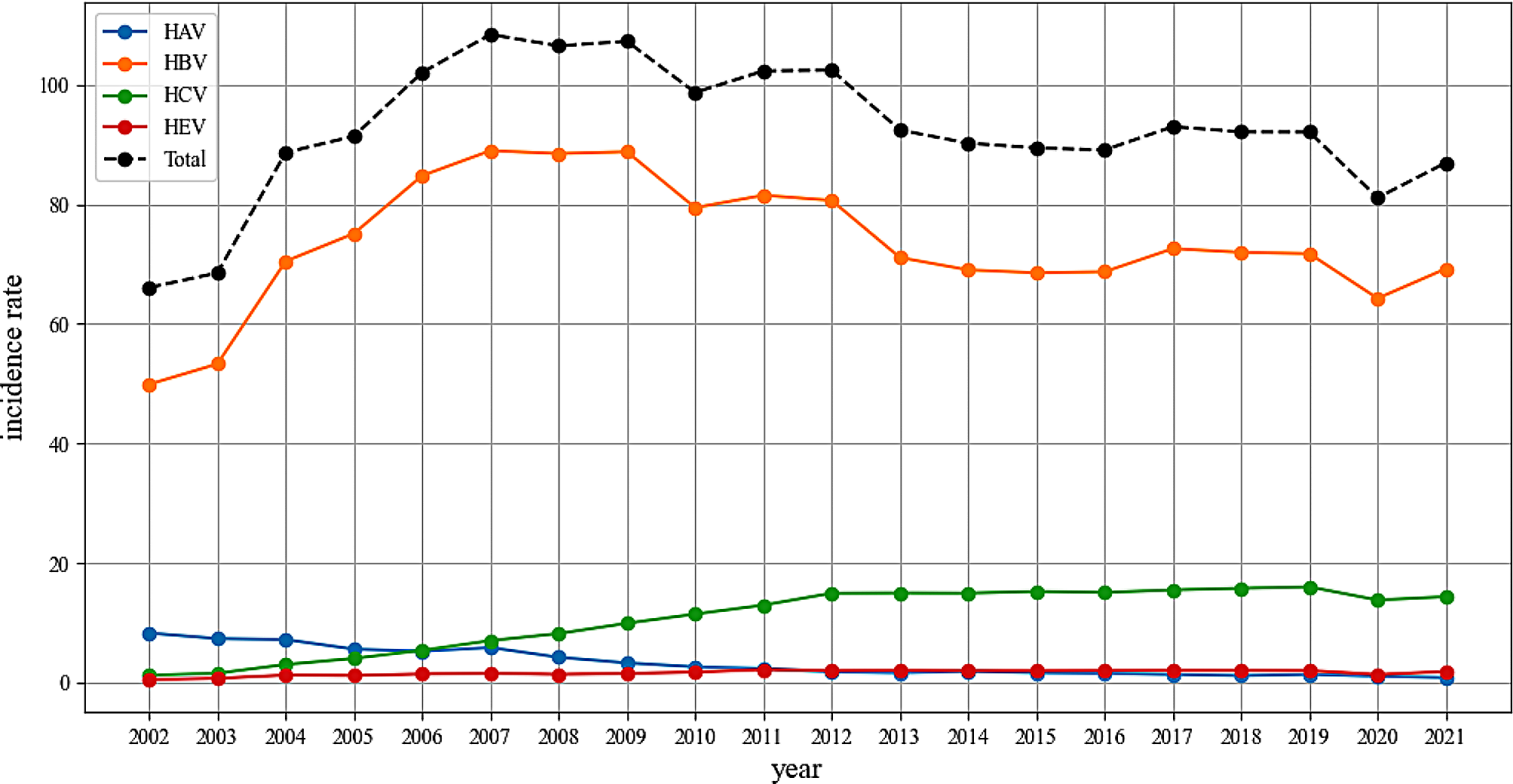
Trends in hepatitis incidence in China from 2002 to 2021

From 2002 to 2021, the annual mean incidence rates of HAV, HBV, HCV, and HEV in China were 3.33, 73.45, 10.79, and 1.65 per 100,000 individuals, with standard deviations 2.41, 10.61, 5.26, and 0.47, respectively. HBV was the most common agents of hepatitis, accounting for nearly 80% of the incidence. As shown in Fig. 1 andS1, the HAV incidence rate slowly declined from 8.29 in 2002 to 0.89 in 2021. The HBV incidence rate increased gradually from 49.91 in 2002 to 89 in 2007, and then generally showed a decreasing trend with slight fluctuations, reaching 69.25 in 2021. The HCV incidence rate increased from 1.24 in 2002 to 15 in 2013, and then fluctuated around 15. The HEV incidence rate increased from 0.51 in 2002 to 2.18 in 2011, and then decreased slightly to 1.85 in 2021.

### Incidence trends based on JoinPoint regression

**Fig. 2:**
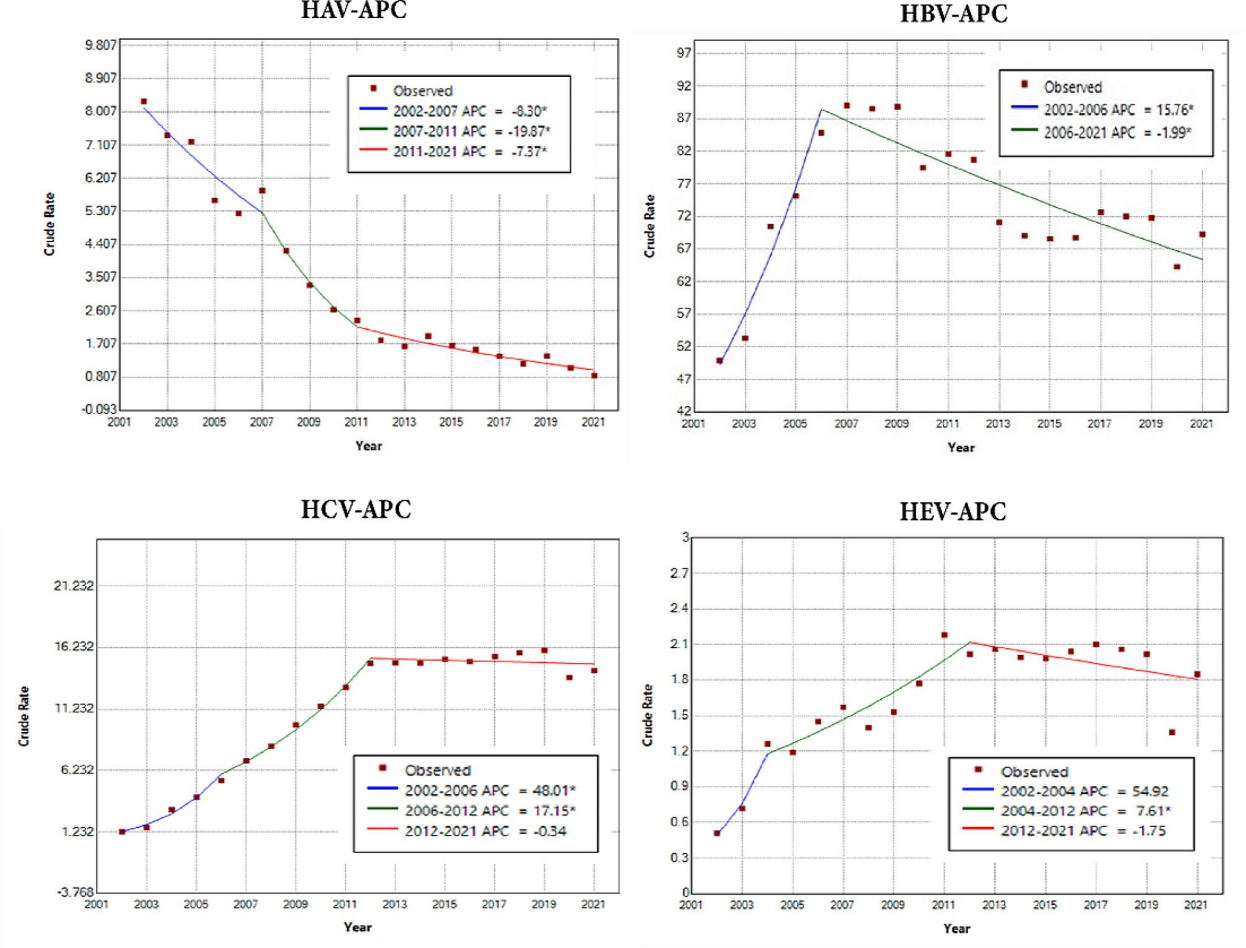
APC diagram of JoinPoint model for each type of hepatitis

The incidence trends of four types of virus hepatitis are shown in Fig. 2 and Table S1. It is observed that the incidence rates of APC of HAV decreased by 8.3% (95% CI: [-11.55%, -4.94%]), 19.87% (95% CI: [-28.33%, -10.40%]), and 7.37% (95% CI: [-9.83%, -4.85%]) in 2002–2007, 2007–2011, and 2011–2021, respectively. Guangdong had the smallest AAPC (-3.13%). Henan had the largest decline in HAV incidence, with an AAPC of -21.56%.

The HBV incidence rate had one breakpoint, split into two trends, with an evident increase in APC of 15.76% (95% CI: [9.04%, 22.90%]) during 2002–2006 and a decrease in APC of 1.99% (95% CI: [-2.66%, -1.31%]) during 2006–2021. Changes in AAPC varied among provinces. The incidence rate in the eastern region showed an obvious upward trend, with AAPC ranging from 2.01 to 7.64%, except in Zhejiang (-5.47%).

Both HCV and HEV showed two phases of increasing trends before 2012. Thereafter they experienced declines of varying magnitude. Geographically, the AAPC of HCV was on the rise in all provinces, with the largest AAPC in Guangdong (19.72%). Regarding HEV, AAPC tended to increase in all provinces except Tianjin (-6.65%).

### The spatial and temporal distribution of viral hepatitis

HAV and HEV exhibited strong spatial autocorrelation during 2002 and 2021, peaking in 2011 with Moran’s I values of 0.675 and 0.428, respectively, suggesting significant clustering in certain regions. P-values below 0.05 in earlier years validate this clustering’s statistical significance. Conversely, HBV and HCV showed a decline in spatial autocorrelation over the years. HBV’s Moran’s I dropped from 0.270 in 2002 to 0.060 in 2021, with rising P-values indicating reduced clustering and a trend towards more uniform geographic distribution. Tibet, Gansu, and Xinjiang frequently emerged as hotspots for both HAV and HBV. The northeastern provinces were the main hotspots for HCV, while HEV frequently appeared in Liaoning as well as coastal regions such as Shanghai and Zhejiang. Tibet was often identified as a cold spot for HBV and HCV. Additionally, Guangdong displayed significant spatial anomalies for multiple types of hepatitis, showing both high-low and low-high patterns, suggesting significant differences in hepatitis incidence rates compared to surrounding areas (Figs. S2 and S3).

**Fig. 3:**
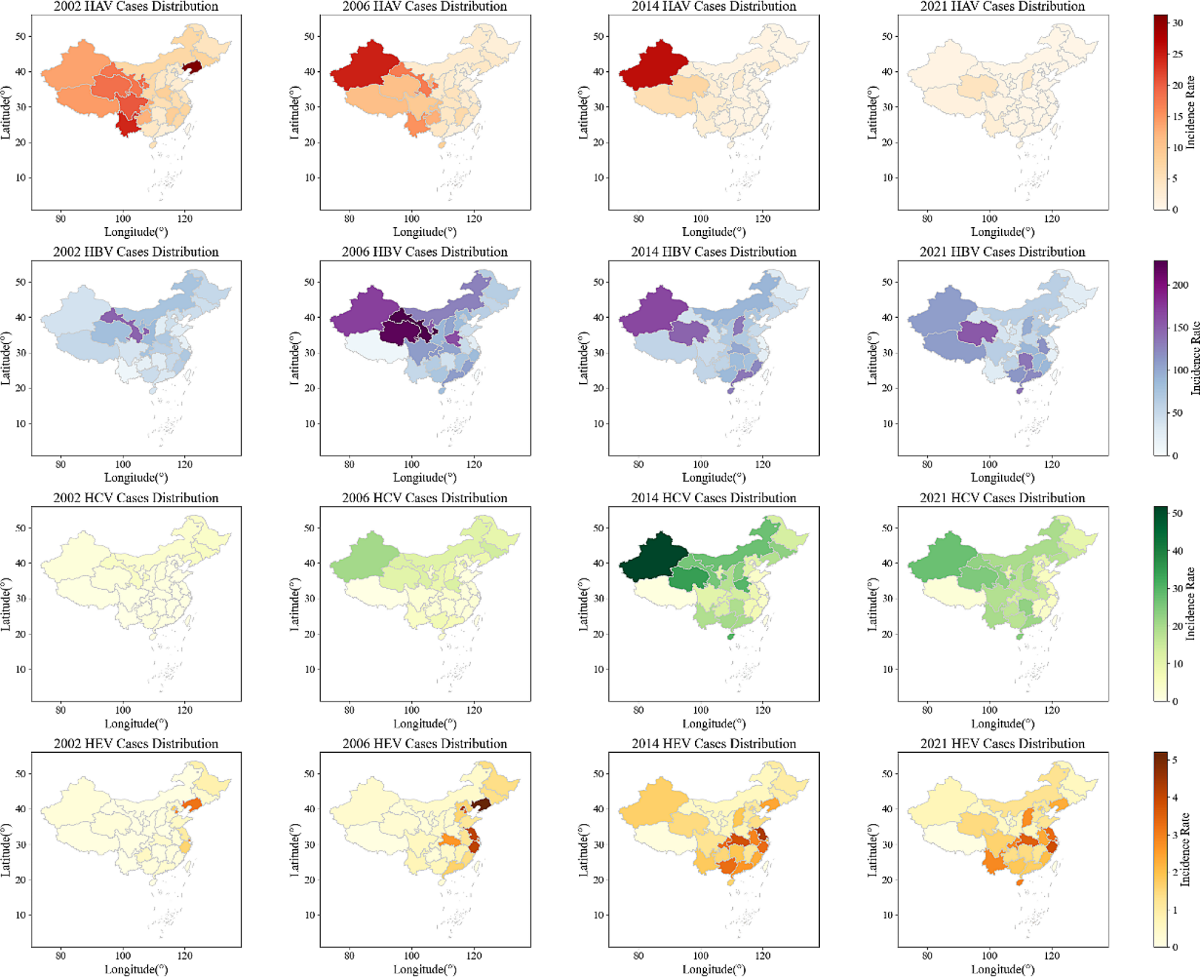
Spatial and temporal distribution of virus hepatitis in 31 provinces in China in 2002, 2006, 2014, and 2021

The spatial and temporal distribution of viral hepatitis is shown in Fig. 3. It is observed that high-prevalence area of HAV located in the Central and Western China, with high - high cluster areas in Xinjiang (15.13) and Qinghai (11.03). The lowest average incidence rate was Tianjin (0.61), followed by Shandong (0.89). The hotspot of HBV has gradually shifted from the northwest to the west and southeast, with the highest incidence rate in Qinghai (202.29), which remained the hardest-hit area in 2021, followed by Xinjiang (154.21). The high incidence of HCV clustered in the northeast and the central as well as parts of the western, such as Xinjiang (33.30) and Qinghai (24.84). The lowest incidence rate was Tibet (1.05), followed by Shandong (2.98). Unlike other hepatitis, the high prevalence areas of HEV were concentrated in the eastern and northeastern region, with the highest incidence rate in Jiangsu (3.57), followed by Liaoning (3.46). The lowest incidence rate was in Tibet (0.11), followed by Ningxia (0.38).

Multifactorial ANOVA showed that there were significant differences and interactions between four regions and most years as far as HAV was concerned. HBV incidence only showed significant regional differences (eastern vs. central and western, northeastern vs. central and western). HCV and HEV incidences showed significant regional and yearly differences (eastern vs. other, central vs. western). HEV incidences showed significant regional and yearly differences (northeastern vs. western).

### Factor extraction via GPCA

The KMO test result was 0.72, and Bartlett’s test probability of significance was 0, indicating that the correlation of the indicators was strong and suitable for principal component analysis. Three principal components with a cumulative contribution rate of 85.198% were selected. Factor model shows the correlations between the factors, in which PA1 contains Beds, HCIs, and HCP; PA2 contains Precipitation, Temperature, and Humidity; PA3 contains COD, SO2, and NH3-N. The indicators ‘Beds’ and ‘NH3-N’ contributed the most (loading factor of 1) to the Hygiene Factor and Pollutant Factor, respectively (Table S2 and Fig. S2).

In terms of the hygiene factor, the eastern and central regions showed better performances, in which Shandong, Henan, Sichuan, and Jiangsu presented high levels, while Ningxia, Tibet, Qinghai, and Hainan had relatively low levels. For pollutant factors, Guangdong, Hunan, Henan, and Jiangsu performed better, while Tibet, Beijing, and Guizhou performed poorly (Table S3).

### Identifying the determinants from a panel fixed-effects regression

The panel data regression with fixed effects was used to obtain the significance and influence of the three principal components in different regions. Table 1 shows the relationship between hepatitis and factors at the national level and in the four major regions. It was observed hygiene and pollutant factor had a significant effect on HAV. High levels of hygiene, low levels of pollutant emissions and better environmental conditions would result in a low incidence rate. For HBV, the health factors substantially reduced the incidence rate in the western and northeastern regions. None of the three factors showed a significant effect in the eastern region. There were no significant regional differences in HCV incidence concerning the meteorological factor. The hygiene factor had little effect on HEV.

**Table 1:** Hepatitis panel fixed effects model test results

HAV	National	Eastern	Central	Western	Northeastern
PA1	-2.8529*** (0.3168)	-1.0198*** (0.1655)	-2.0240*** (0.1635)	-6.1250*** (0.8661)	-2.4540** (0.7731)
PA2	-0.8740 (0.5317)	-1.0834*** (0.2438)	-0.7757** (0.2891)	1.1901 (1.7589)	-0.7883 (0.9202)
PA3	-0.9996*** (0.2199)	-0.3768*** (0.1016)	-0.7848*** (0.1200)	-1.7777* (0.7880)	-0.9460* (0.3774)
HBV	National	Eastern	Central	Western	Northeastern
PA1	-11.2402*** (2.7820)	-1.2200 (2.6289)	-5.4541 (4.3769)	-27.4465*** (6.8648)	-46.1274*** (5.5719)
PA2	-4.5595 (4.6692)	-6.7149 (3.8742)	-3.2532 (7.7396)	8.7622 (13.9422)	-19.5011** (6.6324)
PA3	-2.0910 (1.9311)	-1.1123 (1.6147)	-1.8736 (3.2124)	1.5022 (6.2457)	-10.2014*** (2.7198)
HCV	National	Eastern	Central	Western	Northeastern
PA1	6.0234*** (0.4491)	3.7013*** (0.6456)	6.7324*** (0.7722)	8.0961*** (0.9077)	7.7050* (3.0469)
PA2	1.5600* (0.7538)	1.8611 (0.9514)	-0.9017 (1.3655)	3.2777 (1.8436)	-0.9151 (3.6268)
PA3	2.1345*** (0.3117)	1.0204* (0.3966)	2.6305*** (0.5668)	2.93475*** (0.8259)	3.4474* (1.4873)
HEV	National	Eastern	Central	Western	Northeastern
PA1	0.4040*** (0.0656)	-0.0303 (0.1381)	0.5275*** (0.0937)	0.8472*** (0.0858)	-1.4734*** (0.3375)
PA2	0.2356* (0.1102)	-0.0102 (0.2035)	0.0044 (0.1657)	0.6750*** (0.1747)	-0.2194 (0.4018)
PA3	0.2501*** (0.0456)	0.1394 (0.0848)	0.2459*** (0.0688)	0.25991** (0.0781)	-0.2763 (0.1648)
*N*	527	170	102	204	51

*Note* ***indicates a significance level of 0.001 or less

The meteorological factor loadings were largest among the factors, indicating its big role in the incidence (See Figs. S3 and S4). The regression results of fecal-oral transmitted hepatitis (HAV and HEV) with three meteorological elements were presented as follows.

HAV=-0.0008*precipitation-0.6969*temperature-0.1662*humidity + 25.8059 (in the central region).

HAV=-0.0010*precipitation-0.1420*temperature-0.1012*humidity + 11.8031 (in the eastern region).

HEV = 0.0014*precipitation + 0.1738*temperature + 0.0207*humidity + 3.3616 (in the western region).

Additionally, there was no discernible link between HAV occurrences in the northeastern region and HEV cases, nor with any climatological variables in the central region. Yet, an examination of meteorological factors disclosed a substantial association between HAV incidences and relative humidity. Detailed analysis revealed that relative humidity inversely affected the incidence rates of HAV in the northeastern region. In contrast, a pronounced positive correlation was observed in the central region, where relative humidity levels were positively aligned with the incidence rates of HEV.

The impacts of weather elements on HBV and HCV varied across different regions. In the eastern part, HBV incidences tend to decline with increasing precipitation levels. However, In the central and north-eastern areas, lower temperatures are associated with a reduction in HBV cases, with relative humidity also contributing to this reduction in the north-east, although to a lesser extent than temperature. In contrast, for HCV, higher relative humidity is correlated with an increase in cases in both eastern and central regions. In the northeast, cooler temperatures were associated with a lower incidence of HCV. There is a notable positive correlation between higher levels of precipitation and HCV incidence rates in the west.

## Discussion

We have revealed the spatial-temporal patterns of the viral hepatitis infection and the influence factors in China, by using the JoinPoint model, principal component analysis, and the panel fixed-effects model.

Fecal-oral transmitted hepatitis (HAV and HEV) showed opposite trends and obvious spatial distribution characteristics. Among them, HAV showed a decreasing trend in all three intervals of segmentation. The high incidence of HAV located in the west, and the greatest decline in AAPC of HAV was observed in the central and western regions, such as Henan (AAPC of -21.56%), and Ningxia (AAPC of -17.05%). Similar study found that from 2003 to 2015, the HAV incidence showed a decreasing trend, and the high-high cluster area remained relatively stable and gradually shrunk [15]. The change was attributed to two main factors. First, after the approval and marketing of live attenuated HAV vaccine in 1992, the marketing of inactivated HAV vaccine in 2002, and the incorporation of HAV vaccine into China’s national immunization program in 2009, the number of reported cases of HAV in China decreased from 600,000 cases in 1992 to 14,800 cases by 2019, a record low. Second, China’s overall sanitation has improved significantly, breaking the cycle of viral hepatitis contamination. Improvements in the country’s sanitation facilities coupled with the National Hepatitis A Immunization Program (NHIP) have led to a significant decline in the incidence of acute HAV in the country. By 2019, the incidence rate of acute HAV was 1.9/100,000, which is close to the level of developed countries, indicating that the prevention and treatment of HAV in China has achieved very good results [16]. Due to the relatively underdeveloped economy and poor living conditions in the western part of China, there are greater challenges in ensuring food and water security. Therefore, it remains important to continue to promote access to clean food and water as well as a hygienic living environment to curb the spread and prevalence of HAV in less developed areas.

We found that the prevalence of HAV was modulated by meteorological factors, which showed significant negative relationships with temperature in the central region, and with precipitation in the east. Since HAV is one of the most detected viruses in wastewater, the connection between HAV and rainfall/temperature have been reported in different regions. For example, the reduced incidence rates of HAV were along with the increased precipitation and daily maximum temperature in South Korea [17]. Above 11 °C, there was a decreasing trend in HAV incidence. HAV is usually kept for a long time and is more active at low temperatures [18, 19].

In contrast, the prevalence of HEV was increasing during the study period in China. hepatitis E infections are in most cases episodic cases and occasional foodborne outbreaks [20], which are caused by poor sanitary conditions, including animal and human contamination of water and food. HEV APC increased significantly by 7.61% (95% CI: [2.67%, 12.79%]) from 2004 to 2012, and visualizing the incidence rates of HEV in Chinese provinces from 2006 to 2014. The high-incidence areas gradually shifted from the north to the south. This finding aligns with a recent study [21]. The introduction of targeted vaccination campaigns against HEV is deemed necessary [22].

HBV incidence rates have continued to decline since 2006, largely due to China’s HBV vaccination program [6, 23]. China began to provide free hepatitis B vaccine for newborns in 2002, and free hepatitis B vaccination services for newborns were started in 2005 [24]. From 2009 to 2011, China carried out a vaccination catch-up program for children aged 15 and under, born between 1994 and 2001 nationwide. More than 68 million children were vaccinated as part of this initiative [25]. With the support of national financial funds, the coverage of hepatitis B vaccination for children in China has been effectively ensured. In 2015, the full vaccination rate of 3 shots of hepatitis B vaccine for children in China reached 99.58% [26]. However, in terms of the overall trend of HBV incidence, the total AAPC increased significantly by 1.50% (95% CI: [0.23%, 2.79%]), probably because the main incidence population of HBV in China from 1990 to 2017 has been backwardly shifted to the adult population and the reported incidence rate of this population is indeed on the rise [27]. In addition, young people and adolescents, men who have sex with men, female sex workers, drug users, and mobile populations should receive more attention in the context of increasing mobility and openness to sexual attitudes [28, 29].

Compared to HBV, the HCV infection became more severe from 2002 to 2013. The high prevalence of hepatitis C has been clustered from the northeast to the central and western parts of the country, and in addition, HCV infection has not been given enough attention in China, and many people are not aware of their infections, which may accelerate hepatitis C transmission. Of particular concern is the fact that HCV may be co-infected with HBV and HIV, or both, since both HBV and HCV can be transmitted through body fluid exchange [30].

Eastern and central provinces usually have more developed economic strength and better health facilities, which led to a higher level of health services, including better medical resources, more advanced diagnostic and treatment technologies, and wider vaccination coverage. As a result, the incidences were lower in these areas. In contrast, the western regions faced the challenge of inadequate health services and medical resources, which may lead to higher incidence rates of infectious diseases such as hepatitis. Certain hazardous substances may cause damage to the liver, thereby increasing the risk of hepatitis.

Additionally, we observed significant interactions between hygiene factors and meteorological factors in the HAV, HBV, and HEV models; particularly for HBV, where the interaction coefficient is 11.42. This suggests that the combined impact of meteorological and hygiene factors on health outcomes is not merely additive, but exhibits a synergistic effect. For example, a region with both adequate hygiene resources and favorable meteorological conditions might see health benefits that exceed the sum of the benefits from improving hygiene resources or meteorological conditions alone. However, no significant interactions were found between pollutant factors and either meteorological or hygiene factors.

## Conclusions

The study revealed the trend and spatial-temporal distribution of viral hepatitis in China. The decline in the incidence of HAV and HBV along with vaccination program launched in China. Continued close monitoring is required due to the increasing incidence of HCV. There is no one-size-fits-all program for viral hepatitis intervention in all provinces. An effective response requires a coordinated package of measures that should vary according to the epidemiological dynamics. Formulating prevention strategies tailored to each province was needed regarding to the impact of meteorological factors.

### Electronic supplementary material

Below is the link to the electronic supplementary material.

Supplementary Material 1

## Data Availability

Data is provided within the manuscript. Further inquiries can be directed to the corresponding authors.
