# Supplementary material for: Viral hepatitis in China during 2002–2021: epidemiology and influence factors through a country-level modeling study
Source: BMC Public Health. 2024 Jul 8;24:1820. doi: 10.1186/s12889-024-19318-8 (PMC11232300; doi:10.1186/s12889-024-19318-8)
Supplement: Supplementary file 1 — Supplementary Material 1 [file 12889_2024_19318_MOESM1_ESM.docx]

**Viral Hepatitis in China during 2002-2021: Epidemiology and influence factors through a country-level modeling study**

**Supplementary Material**


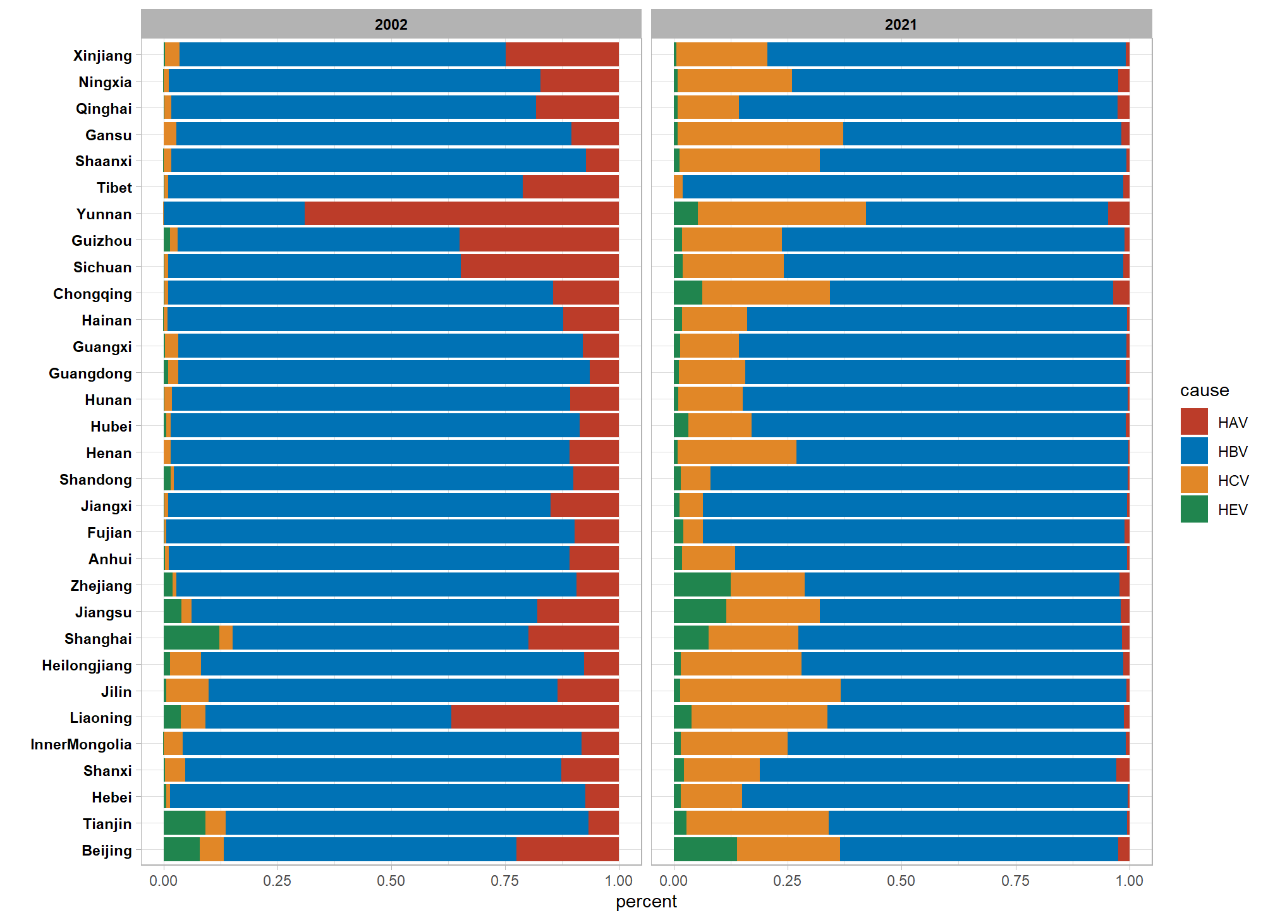


**Fig. S1** Proportions components of four virus hepatitis diseases in China during 2002 and 2021


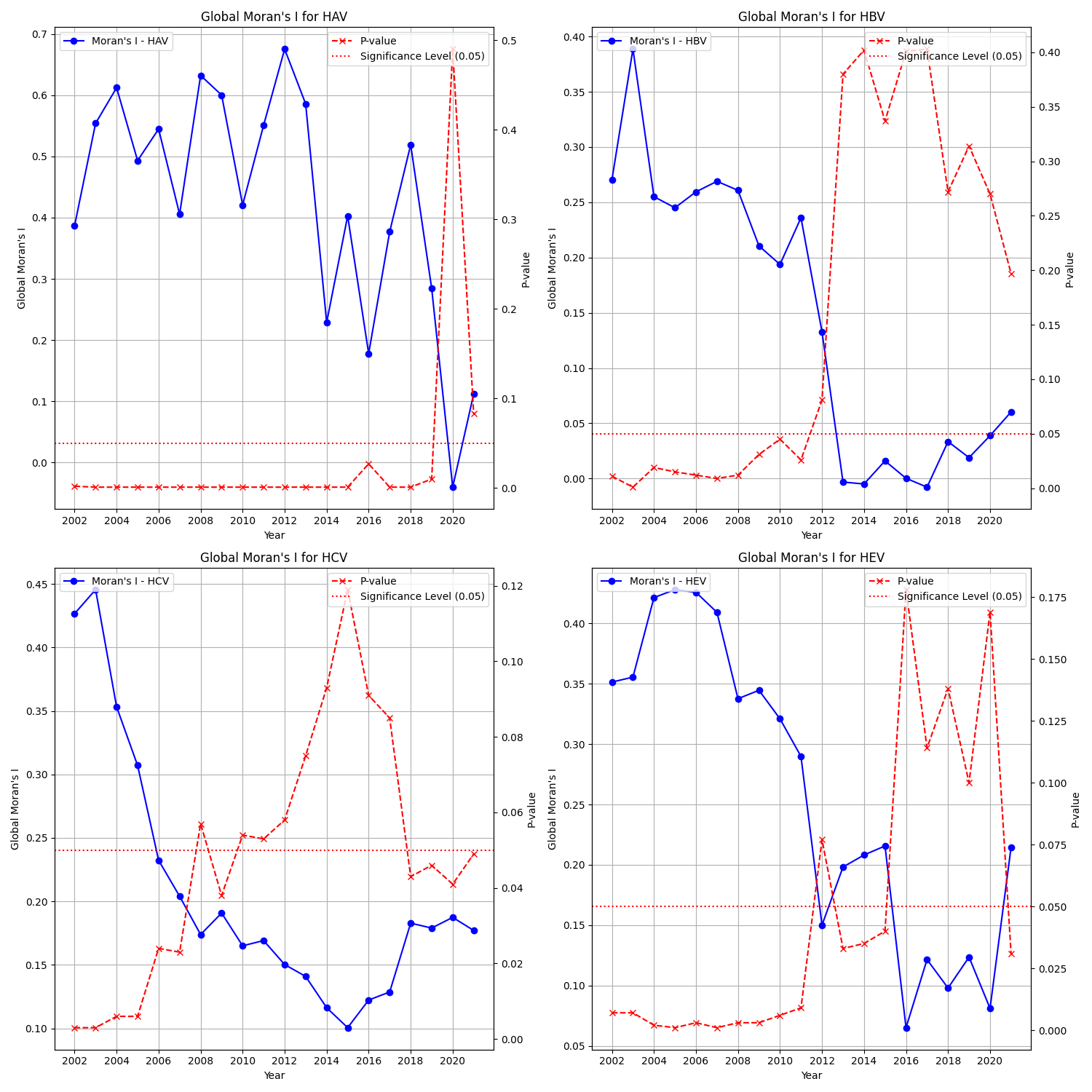


**Fig. S2** Global Moran’s I and p-value for hepatitis from 2002 to 2021


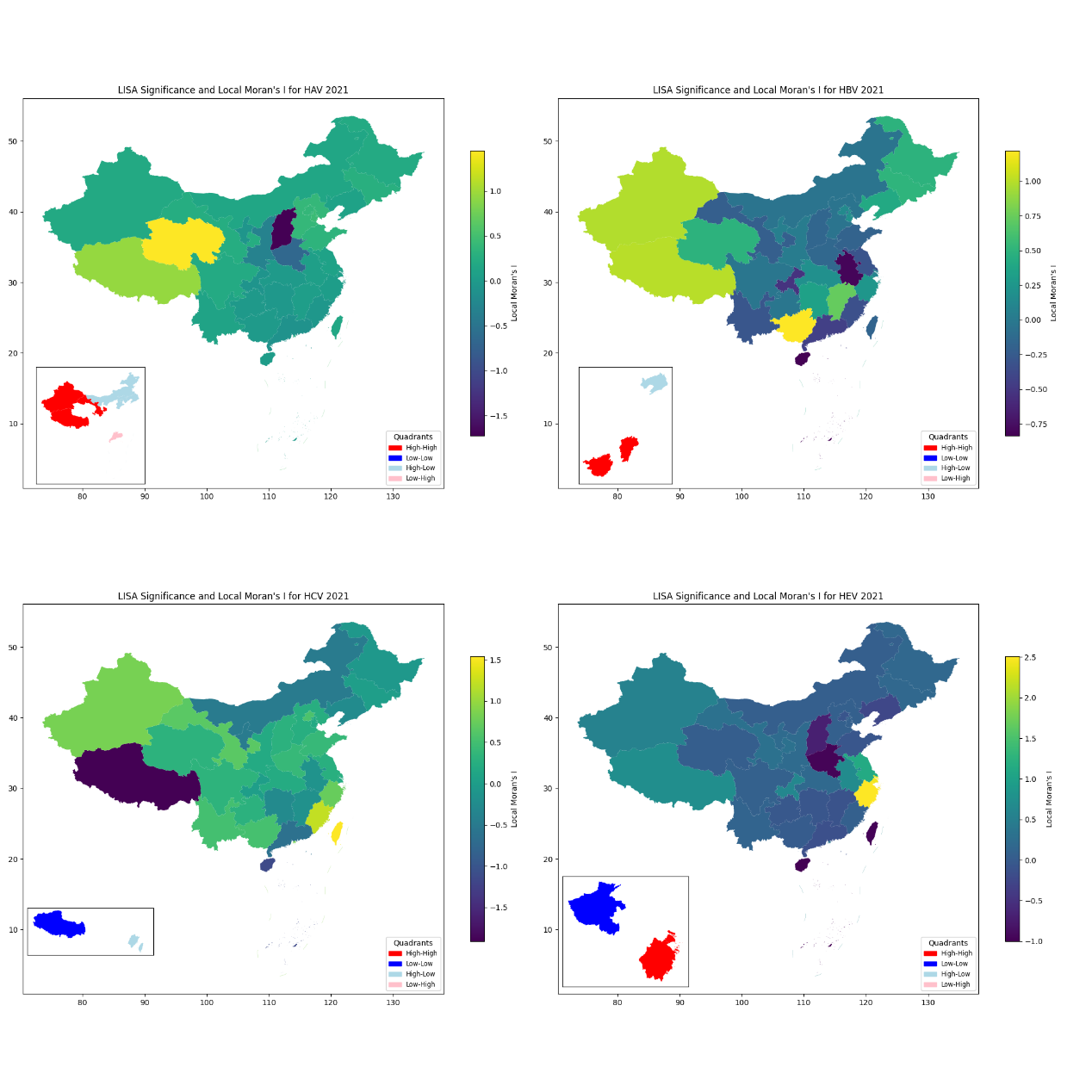


**Fig. S3** LISA map and Local Moran's I map for hepatitis in 2021


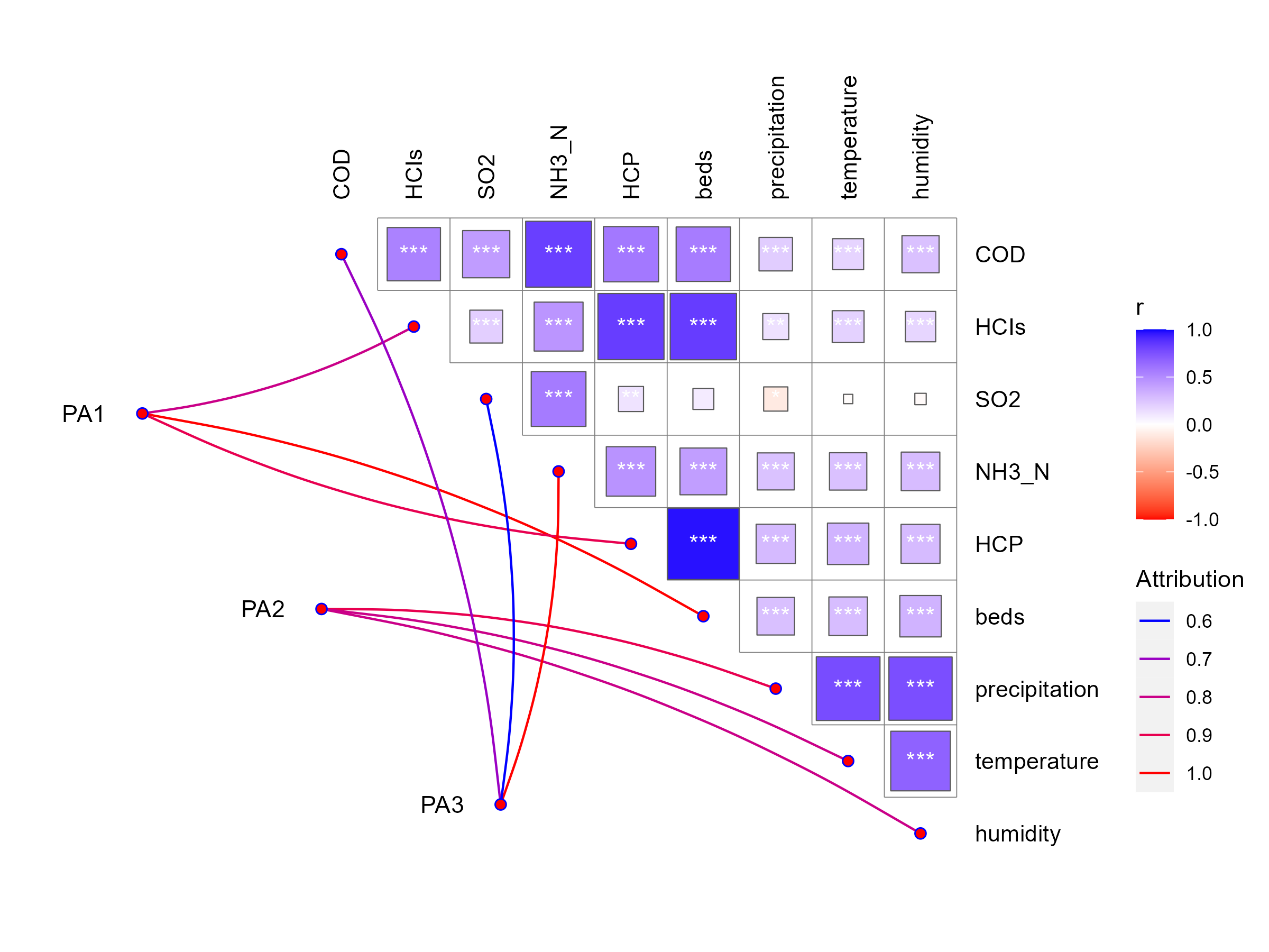


**Fig. S4** The correlations between the factors


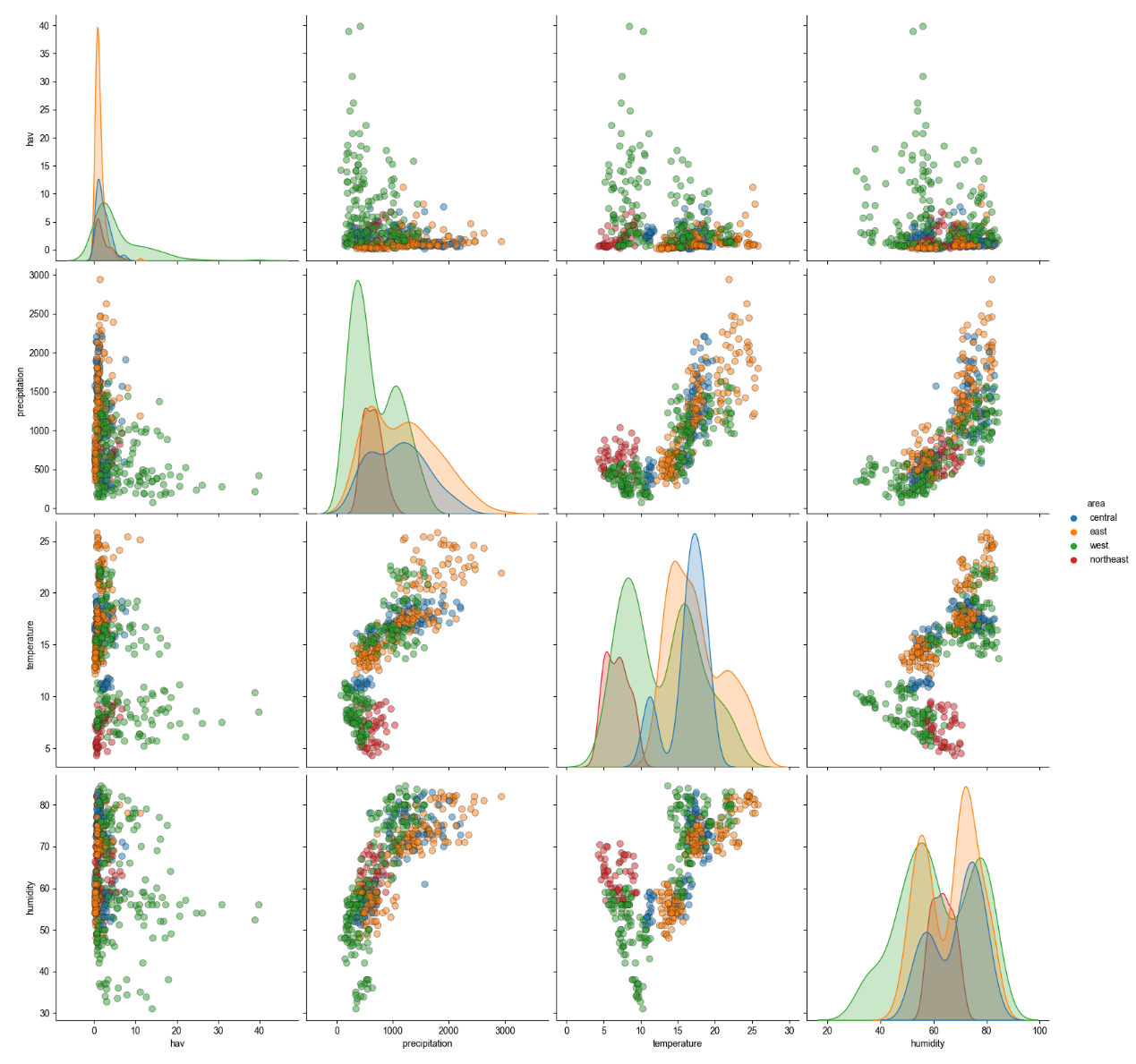


**Fig. S5** Scatter plot matrix of HAV and meteorological factors


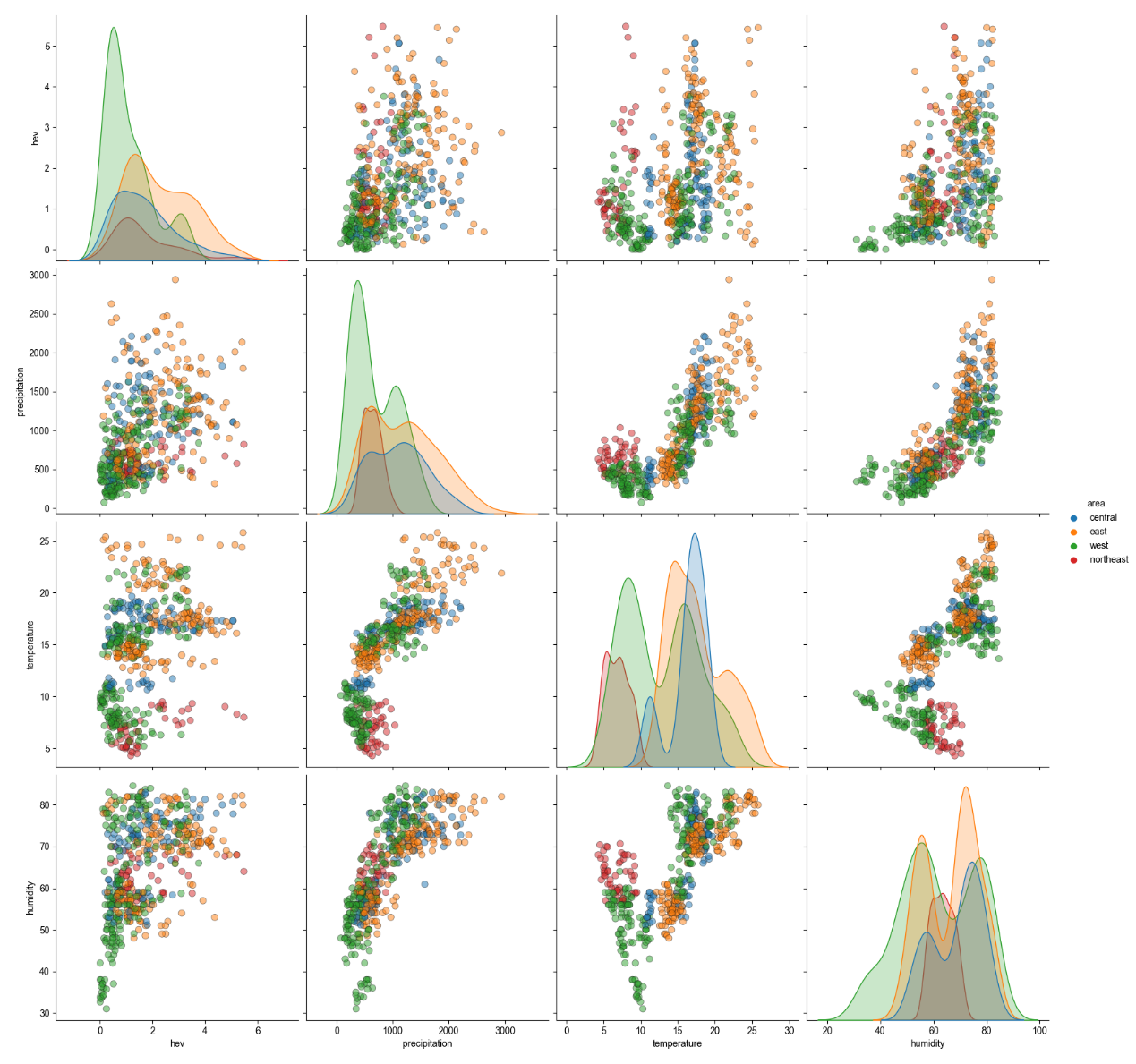


**Fig. S6** Scatter plot matrix of HEV and meteorological factors

Note: Figures S5 and S6 show the scatterplot matrix between HAV, HEV, and meteorological factors (precipitation, temperature, relative humidity). The blue dots represent the central part of the table, the yellow dots represent the eastern part, the green dots represent the western part, and the red dots represent the northeastern part.

**Table S1** AAPC of Hepatitis by Province in China

| Location | HAV-AAPC | HBV-AAPC | HCV-AAPC | HEV-AAPC |
| --- | --- | --- | --- | --- |
| Beijing | -13.3695 | -3.3608 | 2.7433 | -2.4335 |
| Tianjin | -11.1432 | -0.5096 | 11.8515 | -6.6538 |
| Hebei | -12.1568 | 1.3761 | 18.112 | 6.9439 |
| Shanxi | -0.8521 | 8.0453 | 16.1862 | 12.9832 |
| Inner Mongoria | -11.3821 | -0.8878 | 10.0829 | 5.7607 |
| Liaoning | -9.1761 | -0.2492 | 7.1386 | -1.5347 |
| Jilin | -13.1047 | -3.3808 | 3.6413 | 2.6108 |
| Heilongjiang | -11.0289 | -3.2262 | 4.5418 | -0.2784 |
| Shanghai | -9.9942 | 3.0112 | 13.5672 | 0.0445 |
| Jiangsu | -9.7416 | -0.1185 | 13.7264 | 6.0415 |
| Zhejiang | -11.6196 | -5.473 | 11.7732 | 2.4489 |
| Anhui | -11.4352 | 5.1359 | 19.4125 | 14.0423 |
| Fujian | -9.756 | 2.0101 | 13.8371 | 14.1545 |
| Jiangxi | -13.6221 | 3.2514 | 13.9143 | 12.123 |
| Shandong | -9.5227 | 5.3562 | 19.1514 | 1.342 |
| Henan | -21.5626 | -0.6412 | 14.8258 | 8.1142 |
| Hubei | -8.6581 | 1.6835 | 17.4865 | 11.1823 |
| Hunan | -7.6031 | 7.9675 | 22.5218 | 14.0592 |
| Guangdong | -3.313 | 7.642 | 19.7187 | 8.8647 |
| Guangxi | -6.2307 | 4.0564 | 13.8735 | 11.8499 |
| Hainan | -8.1731 | 7.6459 | 16.3661 | 20.1587 |
| Chongqing | -9.7717 | -6.0118 | 16.7448 | 17.2813 |
| Sichuan | -13.5766 | 3.3062 | 18.6223 | 17.7842 |
| Guizhou | -15.1279 | 4.2569 | 19.4649 | 3.7257 |
| Yunnan | -14.5379 | 5.7075 | 44.7746 | NA |
| Xizang | -9.8901 | 3.1215 | 4.8799 | NA |
| Shaanxi | -15.7801 | -5.3383 | 13.9936 | 7.2149 |
| Gansu | -13.9582 | -6.2833 | 8.05 | 10.0023 |
| Qinghai | -7.5516 | 1.5226 | 12.0796 | 9.9277 |
| Ningxia | -17.0509 | -6.1525 | 9.9803 | 6.2756 |
| Xinjiang | -15.2011 | 4.5568 | 14.8946 | 5.0815 |
| total | -10.3944 | 1.5041 | 13.9893 | 7.1044 |

**Table S2** Factor Model

| Factor Name |  |  | Indicator | Attribution |
| --- | --- | --- | --- | --- |
| Hygiene Factor | PA1 | X1 | the number of beds in healthcare institutions (in 10,000) | 1 |
|  |  | X2 | healthcare personnel (in 10,000) | 0.9 |
|  |  | X3 | healthcare institutions (in number) | 0.8 |
| Meteorological Factor | PA2 | X4 | annual precipitation (in millimeters) | 0.9 |
|  |  | X5 | annual average temperature (in degrees Celsius) | 0.8 |
|  |  | X6 | annual average relative humidity (in percentage) | 0.8 |
| Pollutant Factor | PA3 | X7 | ammonia nitrogen emissions (in 10,000 tons) | 1 |
|  |  | X8 | chemical oxygen demand emissions (in 10,000 tons) | 0.7 |
|  |  | X9 | sulfur dioxide emissions (in 10,000 tons) | 0.6 |

**Table S3** Ranking of Health Factors and Pollution Factors by Province in China

| Location | Hygiene Factor | Location | Pollutant Factor |
| --- | --- | --- | --- |
| Shandong | 2.0058 | Guangdong | 1.2947 |
| Henan | 1.7445 | Hunan | 0.9269 |
| Sichuan | 1.3641 | Henan | 0.6157 |
| Jiangsu | 0.9999 | Jiangsu | 0.5845 |
| Hebei | 0.9235 | Sichuan | 0.5343 |
| Guangdong | 0.8743 | Shandong | 0.5302 |
| Hunan | 0.6030 | Hubei | 0.4972 |
| Hubei | 0.3272 | Liaoning | 0.4048 |
| Liaoning | 0.3107 | Heilongjiang | 0.3129 |
| Zhejiang | 0.1523 | Hebei | 0.2977 |
| Anhui | 0.1506 | Anhui | 0.2106 |
| Yunnan | 0.0872 | Guangxi | 0.1682 |
| Shaanxi | 0.0574 | Zhejiang | -0.0213 |
| Shanxi | -0.0033 | Xinjiang | -0.0428 |
| Heilongjiang | -0.0735 | Fujian | -0.0459 |
| Xinjiang | -0.2606 | Shanxi | -0.0579 |
| Inner Mongoria | -0.2823 | Inner Mongoria | -0.1096 |
| Guangxi | -0.2836 | Jiangxi | -0.1321 |
| Guizhou | -0.3243 | Gansu | -0.1369 |
| Jiangxi | -0.3731 | Jilin | -0.1395 |
| Beijing | -0.3839 | Shaanxi | -0.2055 |
| Gansu | -0.3847 | Ningxia | -0.3597 |
| Jilin | -0.4026 | Tianjin | -0.4375 |
| Chongqing | -0.4610 | Qinghai | -0.4444 |
| Fujian | -0.5709 | Shanghai | -0.4454 |
| Shanghai | -0.6667 | Yunnan | -0.4530 |
| Tianjin | -0.8476 | Chongqing | -0.4638 |
| Ningxia | -0.9017 | Xizang | -0.6109 |
| Xizang | -0.9451 | Beijing | -0.6286 |
| Qinghai | -1.0310 | Guizhou | -0.6359 |
| Hainan | -1.4048 | Hainan | -1.0072 |

The general formula for panel regression model, assuming a fixed-effects or random-effects model based on tests like Hausman test, can be represented as:

$Y_{it}=\beta_{0}+\beta_{1}X_{1it}+\beta_{2}X_{2it}+\beta_{3}X_{3it}+\beta_{4}X_{4it}+\beta_{5}X_{5it}+\beta_{6}X_{6it}+\beta_{7}X_{7it}+\beta_{8}X_{8it}+\beta_{9}X_{9it}+u_{it}$

Where:$Y_{it}$ is the dependent variable for i entity at time t.$X_{1it}$to $X_{9it}$are the independent variables for entity i at time t, representing COD, HCIs, SO2, NH3-N, HCP, Beds, Precipitation, Temperature, and Humidity, respectively.$\beta_{1}$to $\beta_{9}$are the coefficients of the independent variables.
